# The Joining of Copper to Stainless Steel by Solid-State Welding Processes: A Review

**DOI:** 10.3390/ma15207234

**Published:** 2022-10-17

**Authors:** Gaurang R. Joshi, Vishvesh J. Badheka, Raghavendra S. Darji, Ankit D. Oza, Vivek J. Pathak, Dumitru Doru Burduhos-Nergis, Diana Petronela Burduhos-Nergis, Gautam Narwade, Gopinath Thirunavukarasu

**Affiliations:** 1 Department of Mechanical Engineering, Marwadi University, Rajkot 360003, Gujarat, India; 2 Fusion Blanket Division, Institute for Plasma Research, Ahmedabad 382428, Gujarat, India; 3 Laxmipati Engineering Works Limited, Surat 394221, Gujarat, India; 4 Department of Mechanical Engineering, School of Technology, Pandit Deendayal Energy University, Gandhinagar 382007, Gujarat, India; 5 Department of Metallurgy and Materials Engineering, The M S University, Baroda 390001, Gujarat, India; 6 Department of Computer Sciences and Engineering, Institute of Advanced Research, The University for Innovation, Gandhinagar 382426, Gujarat, India; 7 Department of Automobile Engineering, Marwadi University, Rajkot 360003, Gujarat, India; 8 Faculty of Materials Science and Engineering, Gheorghe Asachi Technical University of Iasi, 700050 Iasi, Romania; 9 School of Mechanical Engineering, Dr. Vishwanath Karad MIT World Peace University, Kothrud, Pune 411038, Maharashtra, India

**Keywords:** copper, dissimilar, welding, explosive, review, stainless steel, diffusion, intermixing, solubility, processing

## Abstract

Joining immiscible materials such as copper and stainless steel together is a significant concern due to distinct mechanical and metallurgical properties across the joint line, such as melting points, the coefficient of linear thermal expansion, and thermal conductivity. The joint properties of copper to stainless steel welds are in great demand for various mechanical components of the international thermonuclear experimental reactor, ultra-high vacuum system, plan wave linear-accelerator or linac structure, and heat exchanger. These dissimilar-metals joints offer excellent flexibility in design and production, leading to a robust structure for many cutting-edge applications. Hence, the present article reviews the copper to stainless steel joining mechanism under different solid-state processing conditions. The present understanding says that defect-free strong joints between the dissimilar metals are systematically possible. Apart from this understanding, the authors have identified and highlighted the gaps in the research exploration to date. Moreover, a sustainable methodology to achieve a desirable weld of copper to stainless steel depends on favorable processing conditions.

## 1. Introduction—Joining of Copper to Stainless Steel Bimetallic System

Robust and compact structures are the key characteristics demanded for all modern engineering products [1,2,3,4]. The realization of both robustness and compactness in a component is possible only if it is made from multiple materials [5,6,7,8,9,10,11]. Joints of dissimilar materials give different combinations of mechanical and metallurgical characteristics in a product. Thus, bimetallic joints [12,13,14,15,16,17] are necessary, especially in a vacuum [18,19,20,21,22,23] and cryogenic systems [24,25,26,27]. Because of the unique combination of distinct properties, copper/stainless-steel bimetals are in great demand [28,29,30,31,32,33,34]. Stainless steel (SS) combines high strength, low thermal diffusivity, reasonable ductility, and eliminates ductile-to-brittle transitional behaviour. Whereas copper (Cu) embedded high thermal conductivity, better corrosion resistance, and intrinsic absence of ductile-to-brittle transitional behaviour. In this connection, Cu to SS bimetallic amalgamation is the need of the hour for the applications detailed above. Hence, understanding the joining mechanism of Cu to SS under different processing techniques is essential and unavoidable.

**Figure 1:**
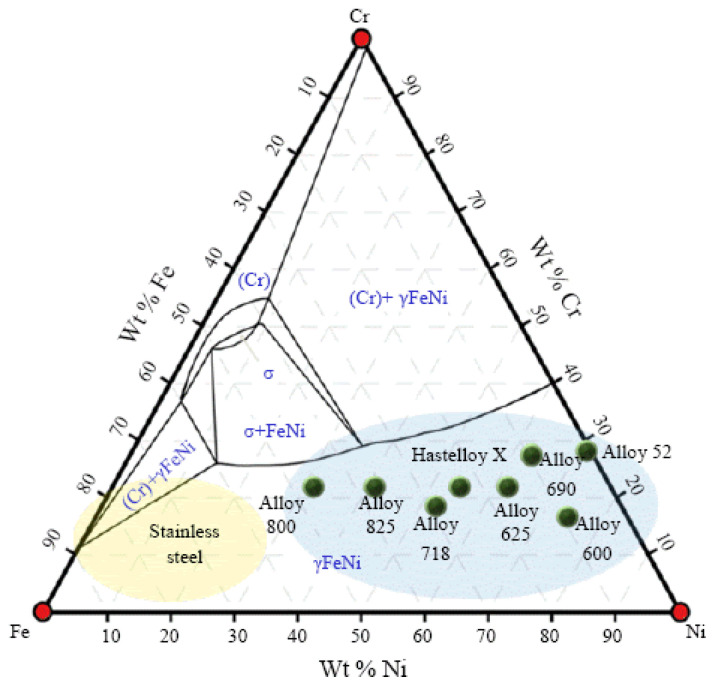
Fe-Ni-Cr ternary phase diagram. Reprinted from Ref. [48]. Copyright © 2022 by The Korean Welding and Joining Society.

**Figure 2:**
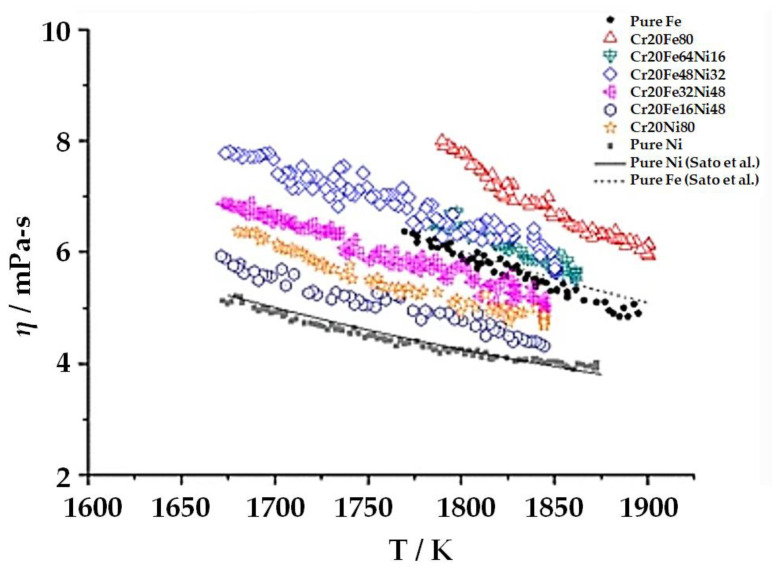
Measured viscosities of various Cr-Fe-Ni alloys as a function of temperature, including pure Fe and Ni [49].

**Figure 3:**
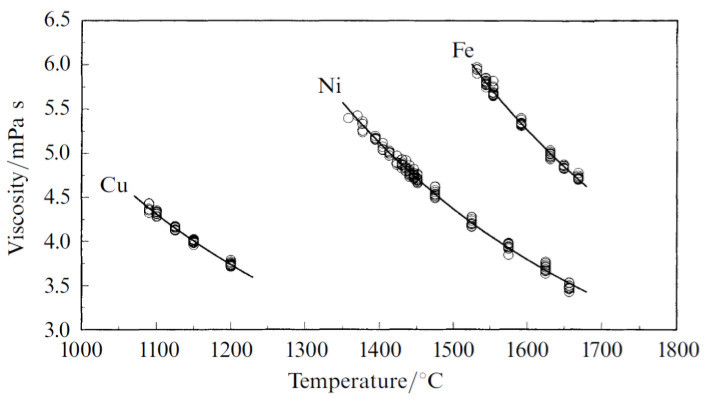
Measured Viscosities of Cu, Ni and Fe by Oscillating Viscometer [50].

However, metallurgical incompatibility (solubility, multiple elements, liquid metal characteristics, and solidification pattern) between copper and stainless steel is significant. The diverse nature of both materials is dominant during fusion welding processes. However, considerable effort has been made to join copper to stainless steel via fusion welding processes [35,36,37,38,39,40,41,42,43,44,45,46,47]. Overall, it is safe to conclude that the metallurgy of copper to stainless steel joints must be the researcher’s focus when welding them with fusion welding processes. Stainless steel is an alloy of Fe-Cr-Ni (along with C, Si, Mn, S, P, and Cu), whereas copper is a metal element. Both have the same face-centred cubic crystal structure. However, that of copper is inherent, while that of stainless steel is a product of the addition of nickel to Fe, which eliminates the ductile-to-brittle transitional behaviour of Fe. Similarly, copper’s corrosion resistance is in-built, while stainless steel it is resistant due to the presence of chromium. Moreover, nickel and chromium improve the mechanical properties. The ternary phase diagram of Fe, chromium and nickel [48] indicated the same (see Figure 1), i.e., that these are responsible for forming stainless steel. The temperatures at which iron (1539 °C), chromium (1907 °C), and nickel (1484 °C) melt are higher than copper (1084 °C). The thermo-physical properties such as viscosity, surface tension, thermal conductivity, and heat accumulation capacity of ferrous-chromium-nickel alloys (stainless steel) are necessary to consider in heat and mass transport processes, i.e., fusion welding. The properties mentioned above (precisely viscosity) lead to complexity in its estimations, measurements, and calculations. Nonetheless, viscosity has been studied (see Figure 2) earlier [49]. The iron’s viscosity improves with the addition of chromium, while pure nickel holds lower viscosity than that of Fe-Cr alloy (overall viscosity of the stainless steel is improved/high). Fe-Cr alloy indicated the highest viscosity (see Figure 2). Similarly, the pure copper’s viscosity during the fusion welding process (since copper’s phase changed from solid to liquid during the welding and again from liquid to solid during solidification, post welding) is very low (see Figure 3) [50]. It signifies the heterogeneous metal flow of copper and stainless steel in a liquid state

Based on the material’s behaviour in a liquid state while fusion welding, proper fluid flow is essential to efficiently weld either metals or alloys. Copper and nickel are the examples that have been welded successfully [51,52,53,54,55] despite vast differences in viscosities (see Figure 3). Mutual solubility plays a vital role in obtaining better macro and microstructural bonding, i.e., even after favourable flow conditions of the materials during fusion welding. However, the complete miscibility of copper and nickel does not guarantee a good joint. The high fluidity of copper and high viscosity of nickel are equally prone to defects. The turbulence created by extremely fluid copper in its liquid state may be exposed to environmental contamination, leading to porosities and cracks. On the contrary, because of the presence of Ni, the excess fluidity might be reduced by the Ni since it has higher viscosity that of Cu. This can produce defect-free joints in case liquid Ni dominates the fluid flow.

Similarly, dense nickel results in a lack of sidewall fusion due to its excess viscous weld pool and movement of the weld pool towards the sidewalls is difficult because of the sluggish weld pool. This means that the weld pool’s stability is necessary to obtain proper formation of the weld pool. This, in turn, is a sign of a sound (defect-free) weld joint. 

**Figure 4:**
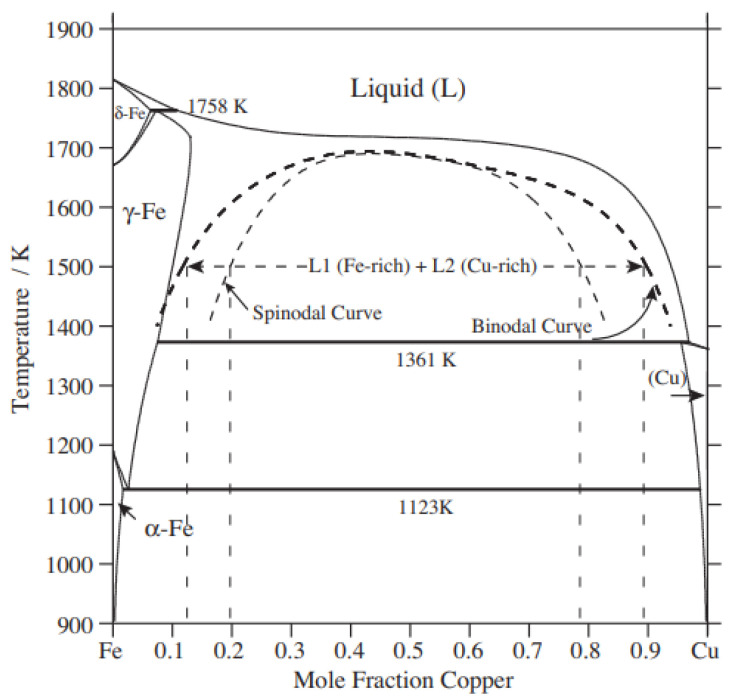
Binary Fe–Cu phase diagram with metastable liquid miscibility gap. Reprinted from Ref. [55]. Copyright © 2022 by Elsevier.

However, the fluid flow of copper and stainless-steel are highly unpredictable due to (1) differences in the flow characteristics of copper, nickel, chromium, and iron; and (2) complete mutual immiscibility (see Figure 4) [50]. Hence, defective welded regions of the Cu/SS joints are unavoidable due to the inherent high turbulence of the immiscible liquid metal flow. This is the reason for solidification cracking or porosity. The above make welding method very difficult, requiring close control over the combination of processes, properties and parameters. However, the efficient joining of copper to stainless steel is still possible by using welding torch oscillation [56,57,58,59,60]. This is a less-explored research area which aims to join the above materials by fusion welding processes. However, it is complicated to weld the above-mentioned materials together using a fusion welding process, i.e., due to the aforementioned fusion-associated challenges. 

Fusion welding of Cu to SS has issues as mentioned above; it is important to report that there is not only a single issue but a series of problems to encounter. To eliminate these challenges, solid-state welding processes are the best choice since they eliminate the issues of fusion welding. In solid-state welding processes, the weld area experiences the temperature above recrystallization, but below melting point [61,62,63,64,65,66,67,68]. In fact, the temperature during the process is generally far below the melting point. In turn, the scientific community should seek the minimal adverse effect of fusion on the joint as well as trying to reduce the heterogeneity in viscosity, miscibility, melting point, thermal conductivity, and thermal expansion. Hence, researchers have considered solid-state joining methods [69,70,71,72,73,74,75,76,77,78,79,80,81,82,83,84,85,86,87,88,89,90,91,92,93,94,95,96,97,98,99,100,101,102,103,104,105,106,107,108,109,110,111,112,113,114,115,116,117,118,119,120,121,122,123,124,125] for the bimetallic joints of Cu/SS. Their works are reviewed in the subsequent sections.

## 2. Solid-State Welding of Copper/Stainless Steel Bimetallic Joint

The fusion of copper and stainless-steel faces several challenges which are not limited to miscibility (mutual solubility) (see Figure 4) and distinct properties across the faying surfaces. The mismatch of thermal induced stresses, complex surface tension, and solidification of multiple elements together demand close control and in-depth analysis while considering joining copper with stainless steel. It is essential to discover an optimal processing window for efficient welding of the dissimilar metals. Solid-state joining techniques avoid the fusion of both copper and stainless steel, which eventually reduces associated challenges due to microstructural differences and differences in physical properties [126,127,128,129,130,131,132,133]. This is why various solid-state joining methods are explored by researchers to join copper/stainless-steel. Here, the joint characteristics are compared and discussed throughout the subsequent sections.

### 2.1. Hot Isostatic Pressing of Copper/Stainless Steel Bimetallic Joint

**Figure 5:**
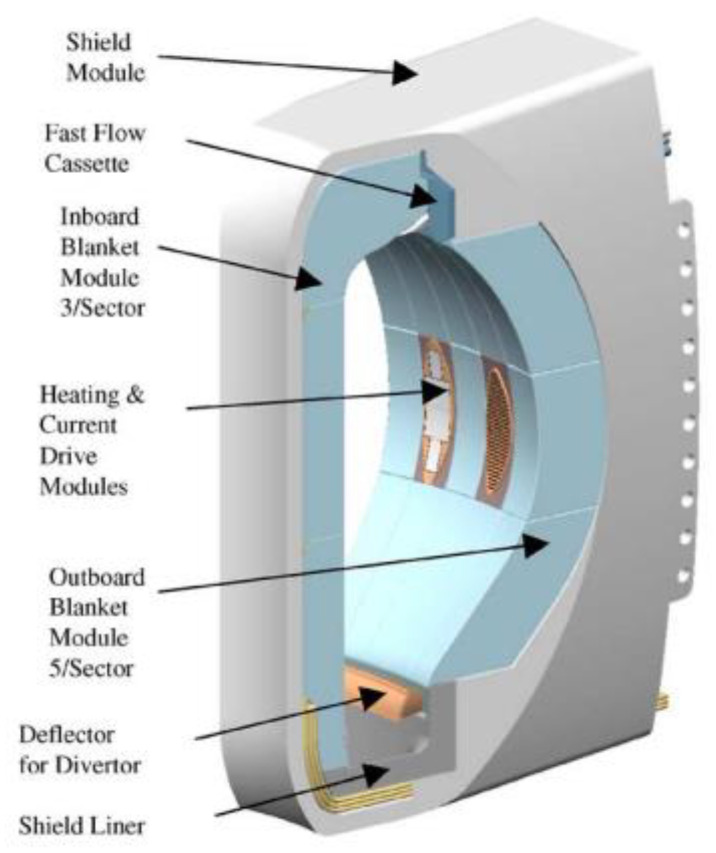
Chamber components: first wall and divertor, blanket and shield. Reprinted from Ref. [136]. Copyright © 2022 by Elsevier.

Hot isostatic pressing (HIP) of copper/stainless-steel bimetallic joints is emphasized to address the need of structural components in the design and realization of an international thermonuclear experimental reactor (ITER), especially for the first wall and divertor [70,91,134,135,136] (see Figure 5) applications. Table 1 summarizes some of the published work in this topic of interest. High heat flux is the primary concern while designing such components [113]. The hypervapotron [122,137,138,139,140,141,142] is a device which can mitigate the effect of heat flux up to 20–30 MW/m^2^ on the entire structure of the fusion reactor. Copper alloy acts as a heat sink [143,144,145,146,147,148] while austenitic stainless steel plays its role as a structural component [123,149,150,151,152,153]. Hence, sustainable joining technology for the mentioned application is necessary. HIP has been selected as a potential joining technique for Cu/SS bimetallic material amalgamation [124]. Extensive research and development (R & D) works were carried out on the Cu/SS joining by HIP (see Table 1). The effect of processing parameters has been assessed on the scale of resulting mechanical and metallurgical properties [124]. The effect of surface polishing and surface roughness on the joining process is discussed in a research paper [124]. In addition to this, the joining of Cu/SS by HIP can be divided into three sections; (1) using interlayer(s) [74,123,124] (2) without interlayer(s) [72,73,75,76,77,123,125,154] and (3) using heat treatment process variations [73,78,79,80,83,91,102,122]. Vital factors considered in the joining are temperature (900–1050 °C), pressure (100–150 MPa) and holding-time (70–240 min) [72,73,75,76,83,122,124]. HIP experiments of Cu/SS bimetallic joint using the above different combinations of levels of the factors resulted in acceptable mechanical and metallurgical properties, such as tensile strength (300–415 MPa), elongation (6–16%) and impact toughness (200–600 J/cm^2^) [124]. Based on the results obtained, it is worthwhile to conclude that the combination of optimum parameters in the range mentioned above can meet the developmental requirements of Cu/SS bimetallic joints for ITER. Thus, HIP is a reliable manufacturing process in the realization of the first wall and divertor components of ITER.

**Figure 6:**
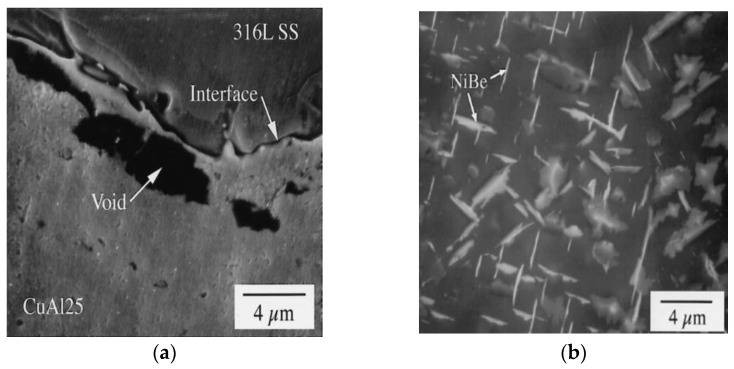
(**a**) Micro voids and (**b**) NiBe precipitation while HIP of Cu/SS joints. Reprinted from Ref. [83]. Copyright © 2022 by Elsevier.

Leedy et al. [83] reported the highest tensile property for the joints of Cu–25%Al and 316 grade SS. In this study, powder-form of Cu–25%Al/316SS showed significant degradation in tensile properties. Cold working of the powder form is not possible, resulting in the restricted interaction of the fine dispersion of oxide particles with high dislocation density. The oxide particles obstruct the dislocation movement, leading to stress concentration, and limits the grain growth. These are the reasons for the reduction in mechanical strength. The oxygen inclusion in the powder Cu/SS joint acts as a diffusion barrier resulting in the reduced intermixing (miscibility) and subsequent weakening of the joint [75]. Dislocation movement and diffusion can be improved by increasing the applied pressure beyond ~102 MPa [72]. On the other hand, base metal strength of copper is degraded due to recrystallization during HIP. Additionally, it was observed that the tensile strength decreases with an increase in the tensile testing temperature. However, the strength of solid-form Cu–15%Al/SS joints exactly match the structural requirement of ITER [122]. The report lacks the discussion on the microstructural evolution for Cu–15%Al/SS joints [83]. Interestingly, for the solid-form Cu–25%Al/SS, powder-form Cu–25%Al/SS and solid-form Cu–15%Al/SS, similar voids (see Figure 6a) were reported near the interfaces. The reason for the voids is precipitation of Fe–Cr–B precipitate. Despite this fact, there is a significant difference in the tensile strength. Interestingly, Cu–Ni–Be/SS does not report any voids, but the tensile strength is lower than Cu–15%Al/SS joint. In contrast, the void formation near the interface is found elsewhere while processing the dissimilar joints of Cu–Ni–Be/SS [76]. The details of the mechanism of void formation were discussed; however, the authors did not provide a proper explanation to correlate the joints’ tensile property and microstructure. Undoubtedly, the shear strength is higher over the temperature range of 25–400 °C for the Cu–Ni–Be/SS joints compared to the Cu-15%Al/SS and Cu-25%Al/SS joints. However, the room-temperature shear strength of the Cu–Ni–Be/SS and Cu–15%Al/SS is similar. The present situation may be attributed to the differences in the base material composition and plate presence as in the case of Ni–Be precipitation. Morphological alteration in Ni–Be precipitation contributed to tensile property degradation attributed to the joint’s cooling cycle (see Figure 6b) [83].

Extensive investigation on Cu/SS bimetallic joints by HIP has been carried out to date, as mentioned in the previous paragraph. The work has been attempted mainly to fulfil the joints’ requirement for applications in ITER [82,91]. From the above conditions explored by different researchers, it is realized that the optimum process condition is 1050 °C, 150-MPa pressure and two hours’ holding time. Post heat treatment is not advisable to achieve the desired property requirements prescribed for ITER. On trying the other possibility, both processing temperature and pressure are brought down [127] using nickel interlayer along with the post-heat treatment to enhance atomic diffusion near the interfacial region. However, optimization of post heat-treatment conditions and the thickness of the intermediate layer need further thorough investigation.

HIP process demands a longer duration time, limited joint geometry, heavy equipment, and a repetitive heating and cooling cycle. Researchers have attempted to investigate the detailed effect of the process parameters on various mechanical and metallurgical properties to develop in-depth knowledge of joint integrity; they found that both understanding of the joint formation and its correlation with process conditions are more complex; however, the application of buffer interlayer in processing Cu/SS joints is not found much in detail. The results obtained for HIP of Cu/SS joint are well accepted as per the requirements of ITER applications; hence, HIP is an excellent candidate for consideration to create the Cu/SS bimetallic joints for an ITER.

### 2.2. Diffusion Bonding for Copper/Stainless Steel Bimetallic Joint 

Unlike the conventional HIP equipment and process, diffusion bonding (DB) reports better joint properties at a lower cost [84,85,86,87,155]. DB can reduce the cost by 40% compared to the traditional HIP [85]. Table 2 highlights the comparative analysis [84,85] between HIP and DB. The entire process is divided into identifying the optimum interlayer material, bond pressure, process temperature, and bonding duration. In comparison with HIP, it is very much clear that the DB needs significantly reduced holding time and bonding pressure, which results in a cost reduction in processing of the joints. Research [89] has shown that the diffusion phenomenon can be significantly increased along the welded interfaces by using additional electrical current supply in a conventional DB procedure. The electric current supply increases the flow of electrons, which directly influences the atomic-diffusion length. The results of electron dispersive spectroscopy (EDS) confirm the higher distribution of atomic species using additional electric current as compared to a conventional DB method. The study of micro-hardness provides evidence that the external current affects the length of diffusion and quantity, but there is no evidence of change in microstructural morphology. The electrical current induces (indirect) heat into the materials and reduces the dislocation movement across the boundaries, resulting in higher rate of diffusion in the materials. A direct correlation exists between the atomic-diffusion length and the tensile properties.

The atomic diffusivity is a function of the temperature [90,156,157]. These studies [90,156,157] reported that the atomic diffusion at Cu/Ni and Ni/SS interfaces increases along with the processing temperature from 800 to 950 °C. The details of Kirkendall voids at both the interfaces are an exact match with those of other published works [83,87,92,155]. The adverse impact of voids on the joints was reported for the joints processed at and above 900 °C. The higher heat energy creates the diffusion gradient, and therefore copper endorses the vacancy adjacent to the interface due to the higher activity of chromium near the region. This leads to the voids. The void significantly appears near the Cu/Ni interface rather than the Ni/SS interface [90,155,156]. The reason is the diffusion gradient. Impulse pressing can reduce the size of the voids [87]. In this context, the optimization of the process parameters can be studied to mitigate or avoid the holes or ultrasonic vibration to enhance atomic diffusion [158]. Undoubtedly, better mechanical properties (see Table 2 [156]) can be achieved by altering the material of interlayer. Furthermore, Au with an Sn-bronze interlayer has also been explored, but the mechanical properties reported were significantly less compared to that of the Au interlayer. The joint efficiency relative to the softer base material for DB of Cu/SS bimetallic joining has been reported [87,89,92,93,155]. Extensive work is demanded in the area to eliminate the voids, increases the mechanical properties at room and elevated temperatures, selection of appropriate interlayer and its thickness, optimum process window in correlation with different joint properties and to improve the electrical resistivity [92]. Nishi et al. [94,95,155,159] carried out extensive work to understand the Cu/SS by DB method.

### 2.3. Explosive Welding for Copper/Stainless Steel Bimetallic Joint 

Explosive welding (EW) has been developed for industrial application since the 1940s, and detailed exploration of the process for different material has reviewed since then [96,104]. Furthermore, detailed discussion on the process is published by Livne et al. [104]. Despite the involvement of explosive material, the process has been put to use for joining of different materials [96], wherein localized plastic deformation over a range of area can be obtained. A few research works are available for the joining of bimetallic Cu/SS [96,97,98,104,125]. The initial study to weld Cu/SS by EW focuses on the joining conditions such as weight ratio $\left(R=\frac{Explosive\;weight}{Flyer\;plate\;weight}\right)$, parallel plate, oblique plate [104]; however, the details on standoff distance are not available in the article. At the same time, the effect of combined standoff distance and R-value is discussed in detail elsewhere [97]. It seems that the work is more inclined towards the impact of R-value on to the joint formation [104]. There is little doubt that the metallographic morphology illustrated away from the detonation point presented dominance of the wavy interface. Furthermore, the degree of intermixing is directly proportional to the weight ratio (R). These results are in conformance with another work published [97]. It was observed that the joints were planar near to the detonation region due to the significant effect of weight ratio (R).

The compositional gradient increases from one wave, defined as molten pockets, to another [104]. The heterogeneous microstructures along the circumference of the molten pockets were mainly attributed to the cooling rate. The cooling rate at the centre of pocket is relatively slow compared to the circumference. However, the heterogeneity in EW is over the entire limited region of interface. Using the EW route for the dissimilar joints, the researchers achieved a tensile strength of 294–372 MPa. Although the strength of base metals is not discussed anywhere else in the paper, the details on the tensile fracture show that the joints’ preferred fracture path was along the copper base metal, which reveals that the dissimilar joints prepared using EW are of far better quality. In addition, it was observed that the mechanical properties increase with standoff-distance and weight-ratio (R) [97] due to the sufficient plastic deformation experienced because of higher impact velocity. The literature [96,97,98,99,100,101,103,104,125] on EW of Cu/SS highlights that the joints have potential to draw more extensive applications. However, a detailed study is needed to explore the effect of temperature on wide range of properties. It is very well established that EW is an efficient process for joining of the bimetals Cu/SS, despite the challenges faced, such as safety due to the involvement of explosions and the degree of heterogeneity in the molten pockets due to cooling rate

### 2.4. Friction Welding for Copper/Stainless Steel Bimetallic Joint 

Researchers have explored friction welding (FW) to join the dissimilar metals, but limited their investigation to pipe joints [160,161,162,163]. FW has shown significant potential to join copper with stainless steel [105,106,107,108]. Joint integrity mainly depends upon the formation of intermetallic along the interfaces. The radial pressure uses the shorter welding time and the sub-melting temperature which eliminates the formation of intermetallic compounds during FW [105,133,164]; hence, sound joints result. The initial study focused on joining 25-mm thick Cu–Cr–Zr to 316 grade SS [109]; wherein, ~2200 RPM, ~353-MPa frictional pressure for 2 s and ~608-MPa upset pressure for 7 sec were employed and the resultant joint between the dissimilar metals exhibited tensile strength greater than the tensile strength of copper (which is ~544 MPa), although the strain rate is unknown. Hence, it is clear that the FW process resulted favourable joint properties of Cu/SS amalgamation. On the other hand, hardness analysis showed the significant effect of heat along the interfacial region of Cu/SS, and in particular the copper-side of the interfacial region. Hardness analysis also indicated that the fracture propagates through the heat affected zone (HAZ) along the copper base-metal. A similar hardness gradient along both the sides of the interfacial region was reported in some other works published [97,98,100,106,107,108]. This clearly indicates limited atomic mobility along both sides of the interfaces for FW procedure compared to other solid-state welding processes such as HIP and DB. Interestingly, tensile properties of 25-mm thick Cu/SS joints were similar to the copper base-metal with lower frictional pressure, upset pressure and RPM [107,108]. In contrast, the study on the heat transfer model for FW of copper to steel has been carried out, and the author [105] has employed a high level of RPM and force. However, the welding duration is in line with work published elsewhere [109].

### 2.5. Friction Stir Welding for Copper/Stainless Steel Bimetallic Joint

The techno-economical aspect of the friction stir welding (FSW) process and its advantages draws the attention of researchers towards the FSW for copper/stainless-steel bimetallic joining. Undoubtedly, the Cu/SS bimetallic joint’s aforementioned critical applications are also one more reason for the industrial and academic attention towards the FSW process. However, the published works on the same materials are limited [110,111,112,114,115,116,117,118,119,165] and summarized in Table 3. Dissimilar material welding via the FSW route needs extra care to obtain sound joints [5,63,166,167,168,169,170]; the reasons are the diversified properties across the joint line [168,169,171] such as melting point, thermal conductivity and coefficient of thermal expansion, flow stress and miscibility (solubility). In addition, the selection of tool material is the most important aspect along with its design, because the joining takes place due to the heat induced (~80%) by tool shoulder [102,103,107,108,109,110,111,161,162,163] and the tool pin governs the stirring action. In a study [120] on the effect of tool shoulder diameter, 18-mm shoulder-diameter with taper tool-pin geometry found suitable for producing quality joints of Cu/SS by FSW. For materials (such as Al) having lower melting range, tool steel [172,173,174,175] is preferred as the tool material. On the other hand, FSW of copper and stainless-steel requires a tool material with a higher melting range, red-hot hardness, and strength; hence, poly-crystalline boron-nitride and tungsten-carbide are preferred to join materials with higher melting points and strength [93,176,177,178,179]. However, tool wear is also one of the major challenges, and limited studies are published on this topic [99,118,180,181,182,183,184]. In fact, tool wear can be avoided by adopting cryogenic treatment [185,186,187,188,189] of the tool materials. Both fixtures and backing plates play a key role in heat balance across the cross-section [190] of the joints. Despite a lower processing temperature than the materials’ melting point, the heat input (from the tool of FSW) must enable the plastic flow of the base materials across the joint line. The heat within the weld area can be retained for a longer time by the judicious selection of proper material for backing plate and fixture; hence, 304L SS is selected for such an application due to its lower thermal conductivity characteristics. On the other hand, sticking of copper with backing plate can also be addressed in the case of high plunge depth. In this case, the researcher has employed the mica sheet beneath the workpiece [110,111]. Similarly, appropriate plastic deformation can also be obtained by applying heat-assisted FSW for Cu/SS joints [121,191,192,193]. The additional heat source, i.e., gas tungsten arc welding torch, ahead of the non-consumable rotating tool softens the base-materials [194,195,196,197]. Hence, the tool of FSW obtained optimal plastic deformation due to the reduced load on the tool, which eventually leads to improved tool life

The microstructural results are a clear reflection of the parametric conditions and the tool design. Particles in diversified sizes and shapes are distributed and became part of the welded area. The interaction between tool pin and SS base-metal (workpiece) is limited due to the tool offset towards the copper side [111,115,116,119,165]. One of the prime reasons for shifting the tool pin towards the copper side is to reduce the load on the tool during processing of the joints, and eventually to increase the tool’s reliability [193]. At the same time, heat balance can also be obtained by shifting the tool towards the copper side; in doing so, plastic flow can be increased by higher demand for the high heat input than SS due to the higher thermal conductivity of copper. Apart from this, researchers [112,115,117] have attempted to offset the tool towards the SS side, wherein sound joints, i.e., free of macro and micro defects, were successfully created. This contrasts with the result presented by another group [107] in which porosity and cracks resulted from ferrous participation in the welded region. On the one hand, this is not a generalized case while joint consolidation depends on the other parametric conditions and mainly on the tool pin’s material flow. On the other hand, the maximum-sized dispersed particle is identified within the weld area when the tool is shifted towards the stainless-steel side. [112,115,117]. This corresponds to the tool shoulder diameter and tool pin dimensions in conjunction with FSW parameters (refer Table 3).

**Figure 7:**
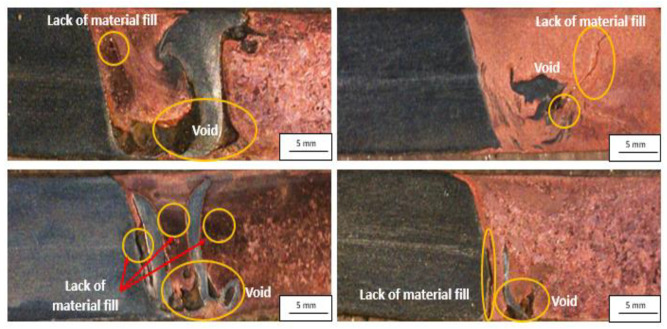
Defects of Cu to SS welded samples via FSW. Reprinted from Ref. [125]. Copyright © 2022 by Springer Nature.

Defects are more likely at the property damping zone. On the other side, the formation of stainless-steel dispersed particles creates the desired intermixing with copper. The report presented the lack of material filling around the scattered stainless steel particle land up with cavity and therefore degraded the joint properties [115] (see Figure 7). A solution to this problem while shifting the tool towards the SS side is available in thermomechanical modelling [110] research. From the simulation work, the higher stress gradient across the faying surfaces leads to the slipping of the material while moving from the leading side to the trailing side of the joint. It will create a material deficiency to fill-up. Hence, the understanding of dispersed stainless steel particle formation and transportation mechanism is of interest to obtain better joint properties. It appears difficult from the literature review. However, it can be correlated with the heat input and the area of tool pin interaction. The higher heat with limited interaction between the SS base material and tool pin (tool pin offset towards the copper side, i.e., softer material region) affected the dispersed particle’s size [110,111,114,115,116,119]. The dispersed particle’s size increases with the high heat input parameter. It is doubtless that the heat input is also responsible for particle dispersion from the SS base material into the welded area. The tool pin shape is also the factor which contributes to the same [114,116].

In this context, the thermomechanical modelling has been reported, and the flow of plasticized material is discussed [110]. Moreover, the SS dispersed particle will be detached from the base material (stainless steel) and then moved beneath the shoulder, where high temperature is experienced. Subsequently, it pushed towards the pin tip at the lower temperature zone. The pin then rotates it and fills the trailing gap during the tool’s forward traverse. The motion of the particle mentioned above also depends upon the material flow speed. Material flow can be predicted through the contact condition between the tool and the deforming sheet. From the simulation results, it is safe to conclude that the tool pin creates the specific rotation flow zone and can be explained by pseudo-sticking state, i.e., governed by the process temperature. The sticking of dispersed stainless-steel particle enables a pin to move it from the advancing side (AS) and keep it at the joint’s trailing side. The discussion is in line with the macro- and microstructure presented elsewhere [110,111,114,115,116,117,118,119,165]. The movement of stainless-steel [119] dispersed particles can be understood in detail in correlation with the welding temperature. The SS suspended particle is situated very near to that of the SS base material interface in each case. It confirms the efficient sticking phenomenon between the tool and the deformed material, i.e., SS. Interestingly, the size of the particle increases with the increase in the processing temperature. Here, the temperature level is predicted by the set of parameters. The SS deformed particle size increases with the increase in the tool rotation from 400 to 500 RPM for both tool traversing speeds: 25 and 50 mm/min. It suggests that the faster impact with SS base material will tear the smaller parts of the SS base. It is wise to conclude that the dispersed SS particles are either from the base material (primary ones) or smaller SS particles (secondary ones) detached from the larger SS particles. The reported model suggests that the detachment of secondary SS particles depends upon the temperature [110]. Moreover, the detailed work is needed to understand the material flow during FSW of dissimilar materials such as copper and stainless steel.

Similarly, to avoid or to eliminate the stretching of stainless-steel particles within the weld area, researchers [112] have recommended the maximum possible offset towards the copper side, wherein the pin is barely touching the stainless-steel base metal. However, the matter is not whether ferrous particle participation is desirable or avoided because good mechanical properties have been reported during each case (Table 3). The hardness graph of the article published [112] confirms the ferrous participation within the joint. On the other hand, one can compare the hardness profile with the tool pin and the stainless-steel interaction. In some cases, the tensile specimen is fractured from the interfaces or the nugget due to the stress concentration over the welded region [111,116]. The heat input is also reported as one reason for fracture at the interface of the weld and stainless-steel base metal. By reviewing the parametric conditions, the higher heat input is responsible for the fracture from the interface [116]. The energy dispersive x-ray analysis and x-ray diffraction results are also in line with the discussion mentioned above [111,116,117,118,119]. Until now, the researcher has exploited the effect of welding speed, RPM, tool pin offset and tool tilt angle on to the joint formation while friction stir welding of Cu/SS (Table 3). Most of the articles reported work on a square butt joint, and the thickness range is 2–6 mm. The lap joint configuration is also exploited [118]. On the other hand, thermomechanical modelling has even been attempted to understand the material distribution and residual stress on the copper/stainless-steel bimetallic joint in FSW [110]. Interestingly, distinct tool shoulder and tool pin material has been applied to weld copper/stainless-steel during FSW [114,117,120,121]. Overall, extensive experimental investigation is needed to know the effect of weld condition on joint formation. 

### 2.6. Another Solid-State Welding for Copper/Stainless Steel Bimetallic Joint

Apart from the above discussed solid-state joining processes, ultrasonic spot welding [198] and the electromagnetic impact technique [199] are studied to join copper with stainless steel, wherein the intermixing region of the joint is comparatively less, which can be confirmed through hardness [199] and microstructural [198] analyses. Interestingly, the microstructural feature discussed for ultrasonic welding is similar to the explosive welding (EW) of copper/stainless-steel. In challenges related to the diversified properties, such as thermal conductivity, reflectivity is eliminated. However, the stacking of materials while ultrasonic welding shows a significant effect on mechanical properties [198]. The higher tensile shear failure load is recorded when copper is placed at the side of the sonotrode. This, in turn, is attributed to the deformation properties of the copper under vibratory conditions. Similarly, the mechanical interlocking is found when copper is joined with stainless-steel by employing the electromagnetic impact technique [199]. However, micro cracks and voids observed in the copper-rich region show evidence of infiltration. Moreover, the articles [198,199] successfully convey the mentioned processes’ feasibility for joining copper with stainless steel. However, it is limited to sheet metals. At the same time, extensive experimental work must be carried out in this area. 

## 3. Conclusions and Future Work

Herein, the literature has been reviewed to understand better the different joint characteristics of dissimilar metals joints copper/stainless-steel (Cu/SS) by various solid-state welding processes, i.e., diffusion bonding (DB), hot-isostatic pressing (HIP), explosive welding (EW), friction stir processing (FSP), friction stir welding (FSW), and ultrasonic welding (UW). Good joining of copper to stainless-steel has been achieved. Moreover, the present review on the research topic allows the following conclusions:

1. Despite obtaining strong weld joints, both DB and HIP face limitation due to part geometry, longer joint formation time, repetitive heating and cooling cycles, and heavy equipment involvement. However, the DB is cost-effective compared to those of HIP. While DB further increases the quantity and length of material diffusion (copper and stainless steel), the electric current application improves in tensile properties. However, work needs to be done to identify the interlayer material and its thickness while adopting DB and HIP processes to synthesize the dissimilar joints of Cu/SS.
2. The ratio of explosive material weight to flyer plate weight and standoff distance are key parameters during EW of Cu/SS. The mechanical properties of the joint increase with the increase in the above-mentioned factors. The microstructural heterogeneity and the extent of diffusion across the joint interface are considered as limitations. The joint interface experiences plastic deformation due to impact energy. The heterogeneity across the length of the joint and within the molten pocket are the main challenges, along with the risk involved in the explosion. Furthermore, diversified dissimilar joint geometries must be explored to increase the application of EW process to join Cu/SS system.
3. The thin layer of intermetallic compounds along the interface during FSW of Cu/SS, present due to limited diffusion compared to DB and HIP, is the essential characteristic of FSW. The tool pin offset on either side of the joint line is the critical factor in deciding the soundness of the Cu/SS joint in FSW. However, offsetting towards the copper side is recommended. The effects of tool pin geometry and shoulder features are not explored during FSW of Cu/SS. Thermal assistance under FSW decreases the load on tool and enhances the joint’s plastic deformation; however, future researchers must establish optimal heat assistance and methods. The material transport mechanism in correlation with the tool interaction with both copper and stainless-steel base materials is of great interest to obtain better joint properties and higher tool life during the FSW of a Cu/SS bimetallic system. While tensile testing, the strain rate has a significant effect on the strength of the joints; hence, strength of the dissimilar joints has to be compared in relation with strain rate of the test.
4. In general, EW, FSW, and UW processes were less explored to join the Cu/SS bimetallic system compared to DB and HIP.
5. Understanding the effect of the geometries (form factor) on the deformation behaviour of both copper and SS are essential to find the optimal operating window for effective and efficient joints of Cu/SS during DB, EW, FSW, HIP, and UW processing routes; hence, a thorough study is targeted on this aspect.

## Figures and Tables

**Table 1:** Summary of literature on the HIP of Cu/SS bimetallic joint.

Substrate	Thickness (mm)	Joint Design	Parameters	Testing Method	Maximum Obtained Properties of the Joint	Reference
Joint 1 CuAl15/316 SS Joint 2 CuNiBe/316 SS Joint 3 CuAl25/316 SS	6/6, 15/10, 15/10	Not reported	Joint 1 & 3 Temperature; 982 °C Pressure: 102 MPa Holding time: 2 h Joint 2 Temperature; 927 °C Pressure: 102 MPa Holding time: 2 h Aged at 500 °C for six h	Tensile test Shear strength test Microstructure SEM EDX AES TEM	UTS (MPa): 290 for joint 1 Shear strength (MPa): 120–125 for joint 2	[89]
DSCu (GlidCop^®^ AL-15 and AL-25)/SS	1.5	Tube-plate joint	Temperature; 980 °C, 1030 °C, 1050 °C Pressure: 150 MPa Holding time: 2 h	Tensile test, SEM Microstructure Charpy impact test Fatigue strength test Fracture toughness test Crack propagation test EPMA analysis Microhardness, Heat flux test	Optimum process temperature: 1050 °C	[153]
Joint 1 GlidCop A125/316 LN SS Joint 2 Cu-Cr-Zr/316 LN SS Joint 3 GlidCop A125/316 SS	Joint 1 25/40	Not reported	Joint 1 Temperature (°C); 920, 1000 Pressure (MPa): 100, 140 Holding time (min) 70, 180 Joint 2 Temperature: 1000 °C Pressure: 140 MPa Holding time: 70 min Joint 3 Temperature (°C); 1050 Pressure (MPa): 150 Holding time (h): 2	Tensile test Microstructure	UTS: 415 MPa Optimum condition Joint 3	[80]
Joint 1 DSCu/316LN SS Joint 2 DSCu/316L SS Joint 3 CuCrZr/316LN SS	Not reported	Not reported	Joint 1 Temperature (°C); 920–1040 Pressure (MPa): 120–140 Holding time (h): 2–4 Joint 2 Temperature (°C); 1050 Pressure (MPa): 150 Holding time (h): 2 Joint 3 Temperature (°C): 920, 1000 Pressure (MPa): 120, 130 Holding time (h): 3, 1 Interlayer: Fe−42%Ni, Ni	Tensile test Microstructure SEM EPMA Impact toughness Fatigue test Fracture toughness	UTS (MPa): 400 % EL: 16 Fracture toughness (J/m^2^): 12.2	[76]
DSCu/316 SS	Not reported	Not reported	Temperature (°C); 800–1000 Pressure (MPa): 100, 120 Holding time (h): 2–4	Tensile test Microstructure Fatigue test EDX	UTS (MPa): 408 JE (%): >100 % EL: 16 Optimum condition Temperature (°C); 980 Pressure (MPa): 100 Holding time (h): 2	[82]
Joint 1 CuNiBe/316L Joint 2 CuAl25/316L	Not reported	Not reported	Fixed parameter Pressure (MPa): 101 Holding time (h): 2 Joint 1 Temperature (K); 1245 Joint 2 Temperature (K); 1255	SEM EDX Microhardness	The significant change in hardness reported	[83]
CuCrZr/316 L SS	7.2/12	Over lap	Temperature (°C); 900 Pressure (MPa): 130 Holding time (h): 2	Tensile test, SEM Charpy impact test Microstructure, EDX	UTS (MPa): 321 Impact toughness (J/cm^2^): 104	[74]

**Table 2:** Summary of literature on DB of Cu/SS bimetallic joint.

Substrate	Thickness (mm)	Joint Design	Parameters	Testing Method	Maximum Obtained Properties of the Joint	Reference
Cu/SS	10	P e joint	Fixed parameter Shielding gas: Ar Temperature (°C): 875 Heating & cooling rate (°C/min);- 20 Pressure (MPa): 3 Holding time (h): 2 Bonding time (min): 30 Type 1 Electrical current (A): 5 Type 2 No electrical current was used	Tensile test Microhardness SEM EDS	UTS (MPa): 170 VHN: 240 Hv Optimum condition Type 1	[95]
Cu/410 SS	3/10	Butt	Temperature (°C); 800, 850, 900, 950 Pressure (MPa): 12 Holding time (h): 1 Heating (°C/min): 30 Ni interlayer: 100 µm	Share strength test Microstructure SEM EDS XRD Microhardness	Share strength (MPa): 145 VHN: 432 Hv Optimum temperature 900 °C	[72,96]
DS Cu/316 SS	20	Butt	Joint type 1 Temperature (K); 1123Interlayer material: Au Interlayer thickness (µm); 20, 60 Bonding pressure (MPa): 4.8, 9.8 Pressed time (h) 1, 2, 3 Holding time (h): 1, 2, 3, 4 Joint type 2 Temperature (K); 1223Interlayer material: Cu Interlayer thickness (µm); 20 Bonding pressure (MPa): 4.8, 9.8 Pressed time (ks) 0.2, 1.4 Holding time (h): 1 Joint type 3 Temperature (K): 1223Interlayer material: Ni Interlayer thickness (µm); 20 Bonding pressure (MPa): 4.8, 9.8 Pressed time (ks) 0.3, 1, 1.1 Holding time (ks): 3.6, 7.2	Microstructure Tensile test Charpy impact test	UTS (MPa): 420 Charpy absorbed energy (J): 25 Optimum condition Temperature (K); 1123Interlayer material: Au Interlayer thickness (µm); 20 Bonding pressure (MPa): 4.8 Pressed time (ks) 3.6 Holding time (ks): 3.6	[154]
Cu/SS	100	Butt	Interlayer: Au (5 µm) + Tin-bronze (500 µm), Tin-bronze (100 µm), Au (100 µm) Temperature (°C); 830, 850, 920, 950 Bonding pressure (MPa): 3 Pressed time (min): 60	Tensile test Microstructure SEM EDS	UTS (MPa): 228 at 0.5 mm/min strain rate Optimum condition Interlayer: Au (5 µm) + Tin-bronze (500 µm) Temperature (°C); 850	[98]
ETP Cu/AIAI 304 SS	20	Butt	Temperature (°C); 700–925 Bonding pressure (MPa): up to 12 Pressed time (min): up to 30 Shielding gas: Ar at 3 bar	Microstructure SEMEDS XRD	Optimum condition Temperature (°C); 800–850 Bonding pressure (MPa): 4–6.5 Pressed time (min): 15–20	[92]
Cu/304 L SS	12	Butt	Interlayer: Ni (0, 12.5, 50 µm) Temperature (°C); 825, 850, 875 Bonding pressure (MPa): 5–20 Pressed time (min): 5, 10, 20	Tensile test Microstructure SEM EDS	UTS: (Mpa): 217 Optimum condition Temperature (°C); 850 Bonding pressure (MPa): 5–20 Pressed time (min): 20 Interlayer thickness (µm): 15	[93]
ETP Cu/ AISI 304 SS	20	Butt	Temperature (°C); 700, 800, 900 Bonding pressure (MPa): 0.2, 0.65, 1.2 Pressed time (min): 5, 15, 30 Shielding gas; Ar	Tensile test SEM EDS	Specific strength or Joint efficiency (µ): 0.8–1 Optimum condition Temperature (°C); 800–850 Bonding pressure (MPa): 10 Pressed time (min): 20–30	[99]

**Table 3:** Summary of current achievements on FSW of Cu/SS bimetallic joint.

Substrate	Thickness (mm)	Joint Design	Parameters	Testing Method	Properties of the Joint	Reference
Cu/316L SS	5	Butt	RPM: 720 Weld speed (mm/min): 16 Tool material: tool shoulder of Mo and pin of WC SD (mm): 22 Shielding gas: Ar Tool pin type: Cylindrical Pin Length (mm): 4.9 Pin diameter(mm): 5	Tensile test Microstructure SEM Microhardness	UTS (MPa): 225.6 JE (%): 85 VHN: 290 Hv	[117]
Cu/316L SS	2	Butt	RPM: 1000, 1500 Weld speed (mm/min): 100, 200, 300, 350, 450, 550 Tool pin penetration (SS side) (mm): 0, 0.6, 1.6 Tool material: WC-14 Co Shoulder (convex) (mm): 25 Pin length (mm): 1.35 Maximum pin diameter(mm): 5.5 Tool pin type: taper	Tensile test Microstructure Micro hardness	UTS (MPa): 267 JE((%): 87 VHN: 60–100 Hv Optimum condition RPM: 1000 Welding speed (mm/min): 300 Tool pin penetration (SS side) (mm): 0	[116]
Cu/304L SS	3	Butt	RPM: 1000 Tool pin offset (mm): −0.6 (steel side), 0, 0.6, 0.9 Welding speed (mm/min) 14–112 Tool tilt angle: 1.5° Tool material: WC Tool pin length: NR Tool shoulder diameter (mm): 18 Tool pin diameter (mm): root is 5 and t is 3 Tool pin type: taper	Tensile test Microstructure Micro hardness	UTS (MPa): 171.3 at 1.5 mm/min strain rate JE (%): 69 % EL: 6.8 VHN: 300 Hv Optimum condition Welding speed (mm/min): 40 Tool pin offset (mm): 0.9 towards Cu side	[118]
Cu/304L SS	2	Butt	RPM: 1000 Tool pin offset (mm): 3 (Cu side) Welding speed (mm/min) 40 Tool tilt angle: NR Tool material: WC Tool pin length: NR Tool shoulder diameter (mm): 10 Tool pin diameter (mm): root is 4 and the t is 2	Tensile test Microstructure Microhardness	UTS (MPa); 216 at 2 mm/min strain rate VHN: 250 Hv	[164]
Cu/304L SS	3	Butt	RPM: 700, 760, 950, 1170, 1450 Tool pin offset (mm): 1 (SS side) Welding speed (mm/min) 25,31.5,40,50,60 Tool tilt angle (°): 1, 1.3, 1.8, 2.3, 2.6 Tool material: Shoulder is of M2 tool steel and pin is of WC Tool pin length: 2.5 Tool shoulder diameter (mm): 22 Tool pin shape: trapezoidal Tool pin diameter (mm): root is 22 and t is 17	Tensile test Microstructure Micro-hardness SEM-EDS	UTS (MPa): 217.2 at 1 mm/min strain rate JE (%): 79 % EL: 20.7 VHN: 250 Optimum condition RPM: 950 Welding speed (mm/min): 40 Tool tilt angle(°): 1.8	[120]
Cu/DSS	4	Butt	RPM: 1000,1200,1400 Tool pin offset (mm) (Cu side): 0,0.5,1 Tool plunge depth (mm): 0.1 Welding speed (mm/min) 20,30 Tool tilt angle (°): 3 Tool material: WC Tool pin length (mm): 4 Tool shoulder diameter (mm): 20 Tool pin diameter (mm): root is 5 and t is 3	Tensile test Microstructure Micro-hardness SEM-EDS XRD	UTS (MPa): 279 at 1 × 10^−3^ s^−1^ strain rate JE (%): 96 VHN: 280 Optimum condition RPM: 1200 Weld speed (mm/min): 30 Tool pin offset (Cu side) (mm): 0.5	[114,115]
CuCrZr/316LSS	6/3	Lap	RPM: 850 Tool plunge depth (mm): 0.5 Welding speed (mm/min) 50 Tool tilt angle (°): 2.5 Tool material: WC-25 Re Tool pin length (mm): 4 Tool shoulder diameter (mm): 16 Tool pin shape: taper spiral Tool pin diameter (mm): root is 7.5, and the t is 6	Tensile test Lap shear testing Microstructure Micro-hardness SEM-EDS XRD	UTS (MPa): 305 %El: 50 Shear test peak load: 19 KN Shear extension (mm): 1.2	[121]
110 Cu/316 SS	6	Butt	RPM: 400, 500 Weld speed (mm/min): 25, 50 Tool pin offset (mm): 2 (Cu side) Tool tilt angle (°): 2.5 Tool material: W-Re Tool shoulder diameter (mm): 16 Tool pin diameter (mm): root is 7 and t is 5 Pin length (mm): 4	Tensile test Microstructure SEM EDX	UTS (MPa): 225 JE (%): 100 VHN: around 375 Optimum condition RPM: 400 Weld speed (mm/min); 50	[122]
C71000/304 SS	2	Butt	RPM: 800, 1000 Weld speed (mm/min): 40, 60, 80 Tool pin offset (mm): 0.75 (Cu side) Tool tilt angle (°): 2.5 Tool material: WC Tool shoulder diameter (mm): 18 Tool pin diameter (mm): 6 Tool pin type: Cylindrical Pin length (mm): 1.8	Tensile test Microstructure SEM EDX XRD	UTS (MPa): 285 %El; 21 JE (%): 84 VHN: around 173	[119]

## Data Availability

Not applicable.
